# Heminested reverse-transcriptase polymerase chain reaction (hnRT-PCR) as a tool for rabies virus detection in stored and decomposed samples

**DOI:** 10.1186/1756-0500-1-17

**Published:** 2008-06-04

**Authors:** Danielle B Araújo, Helio Langoni, Marilene F Almeida, Jane Megid

**Affiliations:** 1 UNESP, School of Veterinary and Animal Science, Department of Veterinary Hygiene and Public Health, Botucatu, SP, Brazil; 2 Center of Zoonosis Control, CCZ, São Paulo, SP, Brazil

## Abstract

**Background:**

The use of methods, both sensitive and specific, for rabies diagnosis are important tools for the control and prophylaxis of the disease. Reverse-Transcriptase Polymerase Chain Reaction (RT-PCR) has been used in rabies diagnosis with good results, even in decomposed materials. Additionally, molecular techniques have been used for epidemiological studies and to gain a better knowledge of viral epidemiology.

**Findings:**

The aim of this work was to evaluate the RT-PCR and hnRT-PCR for rabies virus detection in original tissues stored at -20°C for different periods considering their use for rabies virus detection in stored and decomposed samples. RT-PCR and hnRT-PCR were evaluated in 151 brain samples from different animal species, thawed and left at room temperature for 72 hours for decomposition.

The RT-PCR and hnRT-PCR results were compared with previous results from Direct Fluorescent Antibody Test and Mouse Inoculation Test. From the 50 positive fresh samples, 26 (52%) were positive for RT-PCR and 45 (90%) for hnRT-PCR. From the 48 positive decomposed samples, 17 (34, 3%) were positive for RT-PCR and 36 (75%) for hnRT-PCR. No false-positives results were found in the negatives samples evaluated to the molecular techniques.

**Conclusion:**

These results show that the hnRT-PCR was more sensitive than RT-PCR, and both techniques presented lower sensibility in decomposed samples. The hnRT-PCR demonstrated efficacy in rabies virus detection in stored and decomposed materials suggesting it's application for rabies virus retrospective epidemiological studies.

## Findings

Rabies is a viral, zoonotic and fatal disease, which causes encephalomyelitis in humans and animals. The annual number of deaths worldwide caused by rabies is estimated to be 55 000 and about 10 million people receive post-exposure treatments each year after being exposed to rabies-suspect animals [1].

The disease is caused by a RNA virus that belongs to the *Lyssavirus *genus in the Rhabdoviridae family [2]. All mammals are susceptible to the rabies virus, mainly the *Carnivora *and *Chiroptera *orders.

Developed during the late 1950s, the Direct Fluorescent Antibody Test (DFA) remains the gold standard test in rabies diagnosis because it is fast, with low cost and a great sensitivity [3]. As a single negative test does not rule out the possibility of infection, inoculation tests should be carried out simultaneously with Mouse Inoculation Test (MIT). Alternatively, a monolayer culture of susceptible cells is inoculated with the same material as used for mice [4].

Even so DFA is the gold standard test for rabies diagnosis the sensitivity decreases in decomposed samples [5-8].

MIT is also highly sensitive, but the virus must be viable and the results are achieved after several days of incubation [9]. Additionally, samples in decomposition may present bacteria and/or toxins that disabling the use of the inoculation test [10].

The use of molecular techniques, mainly Polymerase Chain Reaction (PCR) are useful tools in rabies diagnosis [11]. Many works are being made with the PCR for rabies diagnosis, showing that this technique presents highly sensitivity and specificity [12-14].

RT-PCR and hnRT-PCR can detect the rabies virus genome in highly decomposed samples, even when DFA and MIT present negative results, a common situation in countries with tropical weather like Brazil [15-22].

The hnRT-PCR technique allows a sensitive, specific and fast diagnosis for rabies virus, even when samples are in a decomposed state. Additionally, the amplified products can be used in techniques such as sequencing, RFLP, SS-PCR and Multiplex PCR for epidemiological characterization of the virus [20,22]. However, the recommendation for RNA is the storage at -80°C until the moment of its extraction [23], but samples of cerebral tissue are routinely stored at -20°C, in this condition viral RNA can be degraded disabling retrospectives studies.

The aim of this study was to evaluate the Reverse-transcriptase Polymerase Chain Reaction (RT-PCR) and Heminested RT-PCR (hnRT-PCR) techniques for the detection of rabies virus genome in brain samples stored at -20°C (average freezers) for distinct periods and after decomposition for 72 hours in room temperature.

## Methods

It has been analyzed 151 brain samples from different animal species, all suspected of rabies, previously diagnosed for DFA and MIT and stored at -20°C. Samples were 20% homogenate in sterile physiological solution and part of these samples were left at ambient temperature (20–30°C) during 72 hours resulting in decomposed material.

Challenge virus standard (CVS) was used as positive control and non-inoculated mouse brain as negative control.

Total RNA was extracted from the samples by the TRIzol^® ^(Invitrogen) method according to manufacturer's instructions. Reverse transcription was performed with 7 μL of the extracted RNA added to a "mix" containing: 01 mM of dNTP mix, 20 pmols of externals primers P510 and P937, 1 × RT Buffer, 01 mM of DTT and 200 U (units) of MuLV RT^® ^200 U/μl (Invitrogen) enzyme in a final volume of 20 uL. The mixture was incubated for 1 h at 42°C.

The followed RT-PCR and hnRT-PCR protocol and oligonucleotides (table 1) were used according to Soares et al. [18] designed to amplify segments located in the middle of the N gene of rabies virus.

**Table 1:** Oligonucleotides used for the RT-PCR (Reverse-trancriptase Polymerase Chain Reaction (RT-PCR) and heminested RT-PCR techniques.

Primer	Sequence	Sense
P510:	ATA GAG CAG ATT TTC GAG ACA GC	(sense)
P784:	CCT CAA AGT TCT TGT GGA AGA	(anti-sense)
P942:	CCC ATA TAA CAT CCA ACA AAG TG	(anti-sense)

A primary amplification was performed in 2.5 μL of the cDNA added to a "mix" containing 0.2 mM of dNTP mix, 25 pmols of external primers P510, and P942, 1.5 mM of MgCl2, 1 × PCR buffer, 02 U(units) of *Thermus aquaticus *DNA polymerase (Taq DNA polymerase)^® ^5 U/μL (Invitrogen) and ultra pure water in a final volume of 50 μL. The amplification was performed on a MJ Research PCT-200 Thermal Cycler. The following cycling conditions were adopted: initial heating at 95°C/3 min, 35 cycles at 94°C/30 sec., 55°C/30 sec., 72°C/30 sec and a final extending step at 72°C/5 min. The heminested amplification was performed in 2.5 μL of the primary amplification template and external primer P510 and internal primer P784, and the thermal cycles for the heminested assay are the same. PCR products were run in 1.5% agarose gel electrophoresis stained with ethidium bromide and observed under UV light with and photographed. Fragments of 455 base pairs(bp) for RT-PCR and 299 bp for hnRT-PCR were observed.

Statistical analysis was performed by the Kappa coefficient (K) to determinate the concordance between the tests considering the MIT as gold standard test. Sensitivity (S) and specificity (E) were also calculated.

## Results

Out of the 151 analyzed samples, 50 were previously positive for DFA and MIT originating from 18 equines and 32 bovines. Considering the thawed samples, 26 were positive for RT-PCR, with 52% of sensitivity and Kappa coefficient 0.59; and 45 (90% of sensitivity and Kappa coefficient 0.92) when analyzed to hnRT-PCR. Considering the 48 positive decomposed samples, 17 were positive for RT-PCR, with 35% of sensitivity and Kappa coefficient 0.43; and 36 (75% of sensitivity and Kappa coefficient 0.80) with hnRT-PCR.

All previously negative samples (101 samples) for DFA and MIT were negative for RT-PCR and hnRT-PCR (100% of specificity).

Results of the 50 previously positive samples thawed and 48 positive samples decomposed for DFA, MIT, RT-PCR and hnRT-PCR are shown in table 2.

**Table 2:** Comparative results of Direct Fluorescent Antibody Test (DFA), Mouse Inoculation Test (MIT), Reverse-transcriptase Polymerase Chain Reaction (RT-PCR) and Heminested RT-PCR techniques for rabies diagnosis in thawed and decomposed positive samples.

				Thawed		Decomposed	
Samples	Specie	DFA	MIT	RT-PCR	hnRT-PCR	RT-PCR	hnRT-PCR
220-04	Bov	+	+	-	-	-	-
210-04	Bov	+	+	-	+	-	+
218-04	Bov	+	+	+	+	-	+
139-04	Bov	-	+	+	+	IM	IM
107-04	Eq	+	+	+	+	IM	IM
R-5473-00	Bov	+	+	+	+	+	+
R-442-01	Bov	+	+	-	+	-	+
R-298-01	Eq	+	+	-	+	-	+
R-1762-01	Bov	+	+	+	+	+	+
R-366-01	Bov	+	+	+	+	-	-
R-318-01	Bov	+	+	-	-	-	-
R-1760-01	Bov	+	+	-	+	-	+
R-370-01	Bov	+	+	+	+	-	-
R-3386-01	Bov	+	+	-	+	-	-
R-2210-01	Eq	+	+	+	+	+	+
R-3499-01	Bov	+	+	+	+	+	+
R-1001-01	Bov	+	+	-	-	-	-
R-4938-01	Bov	+	+	+	+	+	+
R-441-01	Bov	+	+	-	+	-	-
R-404-01	Bov	+	+	+	+	+	+
R-3388-99	Bov	+	+	+	+	+	+
R-5070-01	Bov	+	+	-	+	-	+
R-207-01	Bov	+	+	+	+	+	+
R-1779-01	Eq	+	+	+	+	+	+
R-5159-01	Bov	+	+	-	-	-	-
R-027-02	Eq	+	+	-	+	-	+
R-2167-01	Eq	+	+	+	+	-	+
R-2575-03	Eq	+	+	+	+	-	+
R-0081-02	Eq	+	+	+	+	+	+
R-0297-01	Eq	+	+	-	+	-	+
R-785-01	Eq	+	+	-	+	-	+
R-3942-02	Eq	+	+	+	+	+	+
R-7124-03	Eq	+	+	+	+	-	+
R-511-02	Bov	+	+	+	+	+	+
R-248-02	Eq	+	+	-	+	-	+
R-235-02	Eq	+	+	-	+	-	+
R-1831-01	Eq	+	+	-	+	-	+
R-3805-02	Bov	+	+	-	+	-	-
R-6429-02	Eq	+	+	-	-	-	-
R-119-02	Bov	+	+	-	+	-	-
R-120-02	Bov	+	+	+	+	-	+
R-299-01	Eq	+	+	+	+	+	+
R-367-01	Bov	+	+	+	+	+	+
R-5093-01	Bov	+	+	+	+	+	+
R-5160-01	Bov	+	+	-	+	-	+
R-4065-01	Bov	+	+	-	+	-	+
R-2056-01	Bov	+	+	+	+	+	+
R-4286-01	Bov	+	+	-	+	-	-
R-6558-02	Eq	+	+	-	+	-	+
515-05	Bov	+	+	+	+	+	+

IM = Insuficient Material DFA/MIT: previously evaluated Samples in bold: false negative results in decomposed materials Bov: bovines Eq: equines

**Figure 1:**
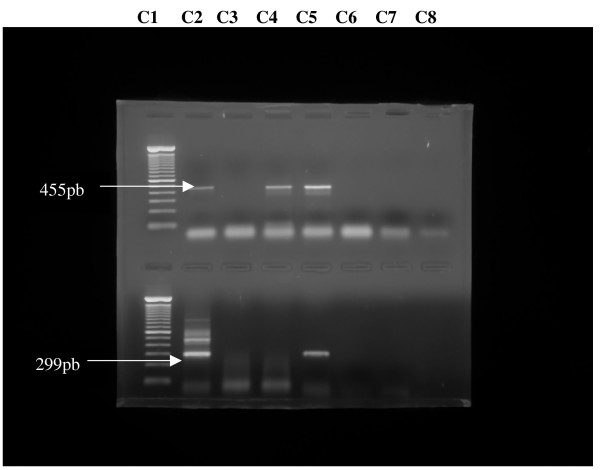
**Reverse-transcriptase Polymerase Chain reaction (RT-PCR) and Heminested RT-PCR (hnRT-PCR) results for detection of rabies virus genome in animal brain samples**. Line 01-C1 – 100 base pairs (bp) ladder; C2 – positive control for RT-PCR with 455 pb; C3 – negative sample (R-441-01); C4 e C5 – positive samples (R-404-01 e R-3388-99); C6 – negative sample (R-5070-01); C7 e C8 – negative controls. Line 02-C1 – 100 base pairs (bp) ladder; C2 – positive control for hnRT-PCR with 299 pb; C3 e C4 – negative samples (R-1001-01 E R-441-01); C5 – positive sample (R-5070-01); C6 a C8 – negative controls.

Figure 1 represents the electrophoresis of samples evaluated to RT-PCR and hnRT-PCR.

The sensitivity of RT-PCR on thawed samples was 52%, while the sensitivity with the association of hnRT-PCR was 90%. These results corroborate previous studies with nested technique for rabies diagnosis; Kamolvarin et al. [14] found 38% of sensitivity for RT-PCR and 100% when considering the nested step.

Considering the Kappa coefficient values, the concordance in thawed samples was 0.59 for RT-PCR and 0.92 for the association with hnRT-PCR; in decomposed samples was 0.43 and 0.80 for RT-PCR and hnRT-PCR respectively, confirming the greater sensitivity of heminested when compared with RT-PCR alone. These better results can be explained by the fact that in heminested assay a second amplification of the template increases the sensitivity being capable of detecting minimum quantities of virus [10].

The analyzed samples were stored from 1999 to 2006. These samples were also evaluated considering the time of storage in order to determine the influence of the freezing time in the observed results. However, no significant differences were observed; in samples from 2001, 50% of the positive ones were positive for RT-PCR, while in the ones from 2002, 33,3% were positive in the same situation. Lopes et al. [20] found a sensitivity of 83,3% in samples stored between the years of 1997 and 2002, the discrepancy in the values may be explained by the temperature of storage between -20 and -70°C, while in the present study this temperature was only of -20°C.

Negative results in the RT-PCR were obtained in 12 decomposed materials, previously FAT and MIT positive. All samples used in this study are from field and were not submitted to viral multiplication; a low viral titer may explain these false-negatives results in the RT-PCR [15,19].

Sensitivity in decomposed samples was lower for both techniques. Decomposed samples were tested to adverse conditions which must be considered in the interpretation of these results, because RNA viruses are much more sensitive to ambient conditions than DNA viruses [11]. The great sensitivity of RNA to RNAses found in the environment and set free during cellular lysis [24] may explain the lower sensitivity in decomposed samples.

The 100% of concordance observed in the 101 negative samples with the results of DFA and MIT demonstrates the high specificity of PCR technique related in many studies for rabies diagnosis [10,12-14].

RT-PCR and hnRT-PCR can detect the rabies virus genome in samples highly decomposed, even when DFA and MIT present negative results [16,17]. When comparing the values founded in the present study with others evaluating samples in the same state of decomposition, hnRT-PCR presented greater sensitivity than DFA and MIT [16,17]. BRITO [8] founded 43,48% and 39,13% of sensitivity for DFA and MIT respectively in samples decomposed for 72 hours, in the present study the sensitivity for hnRT-PCR was 75% for samples in the same conditions.

These results are in concordance with others in decomposed samples with positive results for molecular techniques and negative for DFA and MIT [18,21,22]. Although DFA and MIT are the recommended techniques for rabies diagnosis, RT-PCR can be used as an important tool when samples are not suitable for the standard techniques due to decomposition.

The use of hnRT-PCR can be very useful not only for diagnosis purposes but also using the amplified products for epidemiological classification of the virus [16,18].

Molecular biology techniques, especially hnRT-PCR enabled the detection of rabies virus in brain samples stored at -20°C and decomposed, allowing the retrospective studies and rabies virus epidemiology.

## Competing interests

The authors declare that they have no competing interests.

## Authors' contributions

DBA participated in samples analysis and drafted the manuscript, JM participated in design and coordination of study and helped to draft the manuscript.
